# Growth performance and nutrient digestibility of growing and finishing pigs fed multienzyme-supplemented low-energy and -amino acid diets

**DOI:** 10.1093/tas/txaa040

**Published:** 2020-04-07

**Authors:** Kevin Jerez-Bogota, Cristian Sánchez, Jimena Ibagon, Maamer Jlali, Pierre Cozannet, Aurélie Preynat, Tofuko A Woyengo

**Affiliations:** 1 Department of Animal Science, South Dakota State University, Brookings, SD; 2 Adisseo France S.A.S., Center of Expertise and Research in Nutrition, Commentry, France

**Keywords:** bone mineralization, growth performance, multienzyme, nutrient digestibility, pig

## Abstract

A study was conducted to determine the effects of supplementing corn–soybean meal-based diets with a multienzyme on growth performance, bone mineralization, apparent ileal digestibility (AID) and apparent total tract digestibility (ATTD) of nutrients of growing pigs. A total of 276 pigs (body weight [BW] = 33.99 ± 4.3 kg) were housed by sex in 45 pens of 6 or 7 pigs and fed 5 diets (9 pens/diet) in a randomized complete block design. Diets were positive control (PC); and negative control 1 (NC1) or negative control 2 (NC2) without or with multienzyme. The multienzyme used supplied at least 1,800, 1,244, 6,600, and 1,000 units of xylanase, β-glucanase, arabinofuranosidase, and phytase per kilogram of diet, respectively. The PC diet was adequate in all nutrients according to NRC recommendations and had greater digestible P content than NC1 or NC2 diet by 0.134 percentage points. The PC diet had greater net energy (NE) and standardized ileal digestible amino acids (AA) content than NC1 diet by 3%, and than NC2 diet by 5%. The diets were fed in 4 phases based on BW: Phase 1: 34–50 kg; Phase 2: 50–75 kg; Phase 3: 75–100 kg; and Phase 4: 100–120 kg. Nutrient digestibility and bone mineralization were determined at the end of Phase 1. Overall (34–120 kg BW), pigs fed the PC and NC1 diets did not differ in average daily gain (ADG) and average daily feed intake. Pigs fed NC2 diet had lower (*P* < 0.05) ADG and gain-to-feed ratio (G:F) than those fed PC diet. Pigs fed PC diet had greater (*P* < 0.05) bone ash content and ATTD of P than those fed NC1 diet. The ATTD of GE for PC diet was greater (*P* < 0.05) than that for NC2 diet, and tended to be greater (*P* < 0.10) than that for NC1 diet. Multienzyme interacted (*P* < 0.05) with negative control diet type on overall ADG and AID of GE such that multienzyme did not affect overall ADG and AID of GE for the NC1 diet, but increased (*P* < 0.05) overall ADG and AID of GE for NC2 diet by 5.09 and 8.74%, respectively. Multienzyme did not interact with negative control diet type on overall G:F, bone ash content, AID of AA, and ATTD of nutrients. Multienzyme increased (*P* < 0.05) overall G:F, AID of methionine, ATTD of GE and P, and tended to increase (*P* = 0.056) bone ash content. The ADG, bone ash content, and ATTD of GE and P for the multienzyme-supplemented diets were similar to (*P* > 0.10) PC diet. Thus, NE and digestible AA and P can be lowered by ≤5% in multienzyme-supplemented diets without effects on growth performance and bone ash of pigs.

## INTRODUCTION

The major sources of nutrients in swine diets are plant origin feedstuffs. However, these feedstuffs contain some antinutritional factors, which limit nutrient utilization. Some of the important antinutritional factors present in these feedstuffs include phytic acid and non-starch polysaccharides (NSP). Phytic acid contain P, which is poorly digested by pigs because they do not produce sufficient amounts of phytase to liberate phytic acid-bound P (Woyengo and Nyachoti, 2011). Also, phytic acid reduces digestibility of other nutrients including cations and amino acids (AA) by binding them (Woyengo and Nyachoti, 2013). The NSP are poorly digested by swine and can reduce nutrient availability for digestion and absorption partly by encapsulation and viscosity (Bedford and Partridge, 2010; Bedford and Schulze, 1998; Woyengo et al., 2016). Furthermore, phytic acid and NSP can have negative effects on environment due to increased excretion of unabsorbed nutrients, especially N and P (Woyengo et al., 2008b).

The undesirable effects of phytic acid and NSP can be alleviated through dietary supplementation of phytase and NSP-degrading enzymes also known as NSPases (Cowieson and Bedford, 2009). Corn and wheat grains, and their co-products are the most widely used sources of energy in swine diets in North America and Europe. The most abundant NSP in wheat and corn are arabinoxylans (Choct, 1997; Knudsen, 2014). Wheat also contain some β-glucans (Choct, 1997). Arabinoxylans are composed of backbones of xylans that are substituted mainly with arabinose; arabinose is linked to ferulic acid, which crosslink xylans and lignin (Appeldoorn et al., 2010). Xylanase has been added in corn- and wheat-based diets for pigs with the goal of increasing dietary nutrient utilization by degrading arabinoxylans. However, xylanase had inconsistent effects on nutrient utilization in pigs fed diets that are based on corn, wheat or their co-products. For instance, a few studies (e.g. Ndou et al., 2015) reported improved growth performance of pigs due to addition of xylanase in wheat- or corn-based diets, whereas most studies (e.g. Nortey et al., 2007; O’Shea et al., 2014; Woyengo et al., 2008b) did not report any improvement. The cross-linking of xylans and lignin with ferulic acid can potentially make arabinoxylans more resistant to degradation by xylanase alone. Thus, supplementation of pig’s diets that are based on corn, wheat, or their co-products with a combination of xylanase and arabinofuranosidase can potentially be more effective with regard to degradation of arabinoxylan than supplementation with xylanase alone because arabinofuranosidase can cleave off arabinose units from backbones of xylans, leading to reduced resistance of arabinoxylans to enzymatic degradation. Indeed, supplementation of a multienzyme product that contained xylanase, arabinofuranosidase, β-glucanase, and phytase activities improved nutrient utilization in poultry fed wheat-corn-based diets (Jlali et al., 2018, 2019). However, information is lacking on the effects of the same multienzyme product on nutrient utilization in pigs fed wheat–corn-based diets. Therefore, the objective of this study was to determine the effects of supplementation multienzyme product that contain xylanase, arabinofuranosidase, β-glucanase, and phytase activities on nutrient digestibility, bone mineralization, and growth performance of grow–finish pigs fed corn–wheat–wheat bran–soybean meal-based diets that are low in net energy (NE), standardized ileal digestible AA, Ca, and standardized total tract digestible P.

## MATERIALS AND METHODS

Experimental procedures were reviewed and approved by the Institutional Animal Care and Use Committee at South Dakota State University (#18-015E).

### Experimental Animals

A total of 276 pigs (initial body weight [BW] of 33.99 ± 4.3 kg; Lance-Large White female × Duroc male; Pig Improvement Company) were obtained from the Swine Education and Research Facility, South Dakota State University (Brookings, SD). Pigs were then individually weighed and housed in 45 pens of 6 or 7 pigs. Pens (1.8 × 2.4 m) had fully slated-concrete floors, metal spindle walls (1.0 m high), and solid polyvinyl chloride gates. Each pen was equipped with a cup drinker, and a double-space dry feeder. Room temperature was maintained at 22 ± 2°C throughout the experiment.

### Experimental Diets

Five diets based on corn, soybean meal, wheat, wheat bran, and soybean hulls were fed in this study. The diets included a positive control diet (PC); and negative control diet 1 (NC1) and negative control diet 2 (NC2) without or with multienzyme in 2 × 2 factorial arrangement (Table 1). The multienzyme (a multi-carbohydrase and phytase complex, Rovabio Advance Phy, Adisseo France S.A.S) was added to diets supplying at least 1,800, 1,244, 6,600, and 1,000 units of xylanase, β-glucanase, arabinofuranosidase, and phytase per kilogram of diet, respectively. Enzyme supplementation levels were as per the supplier’s recommendations. The PC diet was formulated to be adequate in all nutrients according to NRC (2012) recommendations. The NC1 diet was the same as the PC diet except that its NE and standardized ileal digestible AA, standardized total digestible P, and total Ca contents were lower than those for the PC diet by 3.0, 3.0, 43, and 18%, respectively. The NC2 diet was the same as the PC diet except that its NE and standardized ileal digestible AA, standardized total digestible P, and total Ca contents were lower than those for the PC diet by or 5.0, 5.0, 43, and 18%, respectively. The reduction in NE value and nutrient content in the NC1 and NC2 diets was achieved by a partial replacement of corn, soybean meal, soybean oil, crystalline AA, calcium carbonate, and monocalcium phosphate in PC diet with wheat bran and soybean hulls. The diets were fed in mash form and in 4 phases based on BW: Phase 1: 34–50 kg, Phase 2: 50–75 kg, Phase 3: 75–100 kg, and Phase 4: 100–125 kg. Titanium dioxide (0.3%) was added as indigestible marker in each diet during the last week of the first phase of feeding.

**Table 1.** Ingredient and calculated chemical composition of the basal diets (%, as-fed basis)^*a*^

	Phase 1: 34–50 kg BW			Phase 2: 50–75 kg BW			Phase 3: 75–100 kg BW			Phase 4: 100–135 kg BW		
Item	PC	NC1	NC2	PC	NC1	NC2	PC	NC1	NC2	PC	NC1	NC2
Ingredients, %												
Corn	55.734	54.033	51.223	60.069	58.059	55.289	61.402	59.623	56.271	65.909	60.757	57.812
Soybean meal	15.211	14.260	13.465	10.351	9.544	9.040	8.814	7.900	7.108	4.953	3.437	2.505
Wheat	10.000	10.000	10.000	10.000	10.000	10.000	10.000	10.000	10.000	10.000	10.000	10.000
Wheat bran	10.000	11.783	15.000	10.000	12.000	14.267	10.000	11.700	15.339	10.000	13.600	16.000
Soybean hulls	2.500	4.500	5.000	3.545	5.471	6.500	4.207	6.289	6.808	4.702	8.438	9.995
Soybean oil	3.225	2.800	2.713	3.000	2.600	2.600	3.000	2.600	2.600	2.300	2.300	2.234
Calcium carbonate	1.083	1.040	1.047	0.998	0.955	0.960	0.875	0.833	0.840	0.689	0.561	0.543
Monocalcium phosphate	1.063	0.415	0.397	0.886	0.236	0.224	0.744	0.096	0.076	0.540	-	-
l-Lysine HCl	0.488	0.480	0.473	0.479	0.470	0.462	0.380	0.380	0.380	0.351	0.349	0.350
dl-Methionine	0.096	0.090	0.086	0.071	0.066	0.062	0.025	0.023	0.021	0.0002	0.0004	0.0004
l-Thronine	0.124	0.121	0.121	0.114	0.111	0.111	0.083	0.085	0.087	0.090	0.094	0.096
l-Tryptophan	0.043	0.041	0.039	0.050	0.047	0.044	0.031	0.030	0.027	0.029	0.028	0.027
Mineral premix	0.150	0.150	0.150	0.150	0.150	0.150	0.150	0.150	0.150	0.150	0.150	0.150
Vitamin premix	0.050	0.050	0.050	0.050	0.050	0.050	0.050	0.050	0.050	0.050	0.050	0.050
Salt	0.234	0.236	0.237	0.238	0.240	0.240	0.239	0.241	0.242	0.237	0.237	0.237
Calculated nutrients												
Crude protein, %	14.735	14.567	14.467	12.943	12.839	12.781	12.249	12.098	12.029	11.108	10.985	10.868
Ether extract, %	5.934	5.493	5.375	5.787	5.367	5.319	5.810	5.390	5.348	5.394	5.371	5.292
NE, kcal/kg	2,475	2,401	2,351	2,475	2,401	2,351	2,475	2,401	2,351	2,475	2,401	2,351
Standardized digestible content of AA, %												
Lys	0.980	0.951	0.931	0.847	0.822	0.805	0.730	0.708	0.694	0.610	0.592	0.580
Met	0.316	0.305	0.296	0.269	0.259	0.251	0.216	0.208	0.202	0.165	0.158	0.154
Met + Cys	0.550	0.534	0.523	0.480	0.466	0.456	0.420	0.407	0.399	0.360	0.349	0.342
Thr	0.590	0.572	0.560	0.509	0.493	0.484	0.456	0.442	0.433	0.400	0.388	0.380
Trp	0.170	0.165	0.162	0.153	0.148	0.145	0.127	0.123	0.120	0.110	0.107	0.105
Total P, %	0.596	0.470	0.487	0.542	0.417	0.428	0.506	0.380	0.398	0.459	0.359	0.371
Digestible P	0.310	0.176	0.176	0.269	0.135	0.135	0.238	0.104	0.104	0.210	0.092	0.093
Calcium	0.660	0.540	0.540	0.590	0.470	0.47	0.520	0.400	0.400	0.460	0.340	0.340
Sodium	0.100	0.100	0.100	0.100	0.100	0.100	0.100	0.100	0.100	1.000	1.000	1.000

^*a*^BW, body weight; PC, positive control diet; NC1, negative control diet 1 with lower in NE, standardized ileal digestible AA, standardized total tract digestible P, and Ca than PC diet by 3, 3, 43, and 18%, respectively; and NC2, negative control diet 2 with lower in NE, standardized ileal digestible AA, standardized total tract digestible P, and Ca than PC diet by 5, 5, 43, and 18%, respectively.

### Experimental Design and Procedure

The five diets were allotted to the 45 pens (9 pens/diet) within a randomized complete block design. Diets and fresh water were offered to pigs ad libitum during the entire period. Pig BW and feed intake were determined by phase to calculate average daily gain (ADG), average daily feed intake (ADFI), and gain-to-feed ratio (G:F). Fresh fecal samples were collected from each pen during the last 2 days of first feeding phase and immediately stored frozen at −20°C for the determination of apparent total tract digestibility (ATTD) of energy and nutrients. At the end of the first phase of feeding, 1 pig (per pen) with BW that was close to the pen average BW was selected, and then euthanized by captive bolt penetration followed by exsanguination. Right and left femurs were excised from each euthanized pig and stored at −20°C for determination of bone ash and bone breaking strength, respectively. Also, contents of lower half of ileum (from 80 cm above ileal-cecal junction to approximately 1 cm above the ileo-cecal junction) were obtained and stored frozen at −20°C for latter determination of apparent ileal digestibility (AID) of energy and nutrients.

### Sample Preparation and Analyses

Femurs for determining bone ash were defleshed by autoclaving at 121°C for 30 min, cleaned and subsequently dried in an oven at 135°C for 2 h. Fat was extracted from the dried bones using petroleum ether (E139-4, Fischer Scientific, Pittsburgh, PA) as solvent in a Jumbo Soxhlet extraction apparatus (Chemglass Life Sciences, Vineland, NJ), afterward the samples were left in a fume hood for 24 h to allow the petroleum ether to evaporate. Femurs were then dried in an oven at 135°C for 2 h to determine their fat-free weight, and ashed at 600°C in a muffle furnace for 12 h for the determination of bone ash. Femurs for determining bone breaking strength were defleshed by scraping muscle tissues from the bones using kitchen knives. Maximal breaking load was measured using an MTS Insight 5 equipment (MTS, Eden Prairie, MN, USA) at room temperature by subjecting each bone to a 3-point bending test (Tunrer and Burr, 1993). Force was applied to the center of the bone held by supports 3.3 cm apart. The crosshead speed was set at 50 mm/min and the sample rate was 10 points/s. Final strength was determined from load–displacement curves.

Fecal samples were pooled by pen and air-dried in an oven at 60°C for 4 d; whereas ileal digesta samples were freeze–dried. The dried fecal and ileal digesta samples together with diet samples were ground through a 0.75-mm screen in a centrifugal mill (model ZM200; Retsch GmbH, Haan, Germany). The ground samples were analyzed as follows: Phase 1 diets for DM, gross energy (GE), N (N × 6.25 = CP), P, AA, NDF, ADF and titanium contents, and for xylanase and phytase activities; Phases 2–4 diets for DM, GE, and N; ileal digesta for DM, GE, NDF, ADF, AA, P and titanium contents; and feces for GE, CP, NDF, ADF, Ca, P, and titanium contents. The samples were analyzed for DM by oven drying at 135°C for 2 h (method 930.15), CP by a combustion procedure (method 990.03), as per AOAC (2012); and for ADF and NDF (Van Soest et al., 1991) on an Ankom 200 Fiber Analyzer (Ankom Technology, Fairport, NY). Samples were analyzed for AA (method 982.30 E [a, b, and c]; AOAC, 2012) at the University of Missouri Experiment Station laboratories (Columbia, MO). The GE was analyzed using an adiabatic bomb calorimeter (model 1261, Parr Instrument Co., Moline, IL). Titanium dioxide in samples was determined by spectrophotometry (model Spectra MAX 190, Molecular Devices, Sunnyvale, CA) at 408 nm after ashing at 525°C for 10 h (Myers et al., 2004). The P content in samples was analyzed according to the vanadate colorimetric method (method 946.06; AOAC, 2012) using a spectrophotometer (model Spectra MAX 190, Molecular Devices, Sunnyvale, CA). The Ca content in samples was analyzed by inductively coupled plasma–optical emission spectrometry (ICP–OES; method 985.01 A, B, and C; AOAC, 2012) after wet ashing samples (method 975.03 B(b); AOAC, 2012). Xylanase and phytase activity in experimental diets were analyzed by the Laboratory of Adisseo (Commentry, France). Xylanase activity was determined according the method described by Cozannet et al. (2017). One visco-unit of endo-1, 4-β-xylanase activity is defined as the amount of enzyme that hydrolyzed by the substrate (wheat AX); one such unit reduces solution’s viscosity, resulting in a change in relative fluidity of 1 arbitrary unit per min per ml (or per g) under the conditions of the assay and pH 5.5 and 30°C. Phytase activity in experimental diets was determined according the standard method (ISO3024, 2009). One phytase unit (FTU) is the amount of enzyme that releases 1 µmol of inorganic orthophosphate from sodium phytate substrate per minute at pH 5.5 and 37°C.

### Calculations and Statistical Analysis

The AID and ATTD values of the diets were calculated using the indicator method (Stein et al., 2007), using the following equation:

$$\begin{array}{l} \begin{array}{ll} & AID\;\;\;or\;ATTD,\;\;\%\;=[1-(Nutrient_{digesta}/Nutrient_{diet}),\\ & \times (Marker_{diet}/Marker_{digesta})]\times 100 \end{array} \end{array}$$

where Nutrient_digesta_ is the nutrient concentration in the ileal digesta or feces (% DM); Nutrient_diet_ is the nutrient concentration in the diet (% DM); Marker_diet_ is the titanium concentration in the diet (% DM); and Marker_digesta_ is the titanium concentration in the ileal digesta or feces (% DM).

Data were subjected to analysis of variance using the MIXED procedure of SAS (SAS Inst. Inc., Cary, NC). The pen was considered as the experimental unit. The model included diet, sex, diet × sex interaction, and initial BW, which was a covariate. Period was the repeated term in models involving time. Means were separated by the probability of difference in order to compare PC diet with other diets. Main effects of NC diet type and multienzyme and their interactions were determined. To test the hypotheses, *P* < 0.05 was considered significant. If pertinent, trends (0.05 < *P* ≤ 0.10) are also reported.

## RESULTS

The analyzed CP values in the diets in Tables 2 and 3 were similar to the calculated CP values in the diets in Table 1. The mean endogenous phytase activity in PC, NC1, and NC2 diets was 406 U/kg. Addition of phytase to the NC1 and NC2 diets resulted in its increased activity values by margins similar to those that were anticipated. The xylanase activity was undetectable in the PC, NC1, and NC2 diets. The analyzed xylanase activity values in multienzyme supplemented NC1 and NC2 diets were similar to the anticipated values.

**Table 2.** Analyzed composition of phase 1 diets as-fed

	Diets^*a*^				
Item	PC	NC1	NC2	NC1 + E	NC2 + E
Dry matter, %	88.5	88.9	89.3	89.1	89.3
Gross energy, kcal/kg	3,973	4,052	4,067	3,990	4,053
Crude protein, %	14.38	14.54	14.81	14.04	14.00
Neutral detergent fiber, %	12.88	15.26	18.25	14.21	17.85
Acid detergent fiber, %	4.50	6.48	7.10	6.00	6.72
Ca, %	0.66	0.52	0.40	0.53	0.37
P, %	0.60	0.51	0.54	0.53	0.50
Indispensable AA, %					
Arg	0.80	0.87	0.83	0.78	0.79
His	0.35	0.38	0.37	0.35	0.35
Ile	0.53	0.56	0.54	0.53	0.51
Leu	1.15	1.19	1.11	1.12	1.04
Lys	0.93	0.97	0.97	0.94	0.93
Met	0.32	0.29	0.27	0.32	0.29
Phe	0.63	0.66	0.63	0.62	0.6
Thr	0.59	0.62	0.59	0.59	0.6
Trp	0.22	0.23	0.22	0.2	0.21
Val	0.63	0.67	0.66	0.63	0.62
Dispensable AA, %					
Ala	0.68	0.72	0.68	0.67	0.64
Asp	1.17	1.25	1.18	1.16	1.09
Cys	0.27	0.27	0.27	0.26	0.27
Glu	2.53	2.61	2.53	2.42	2.51
Gly	0.58	0.64	0.64	0.59	0.62
Pro	0.91	0.92	0.9	0.88	0.88
Ser	0.59	0.63	0.58	0.57	0.56
Tyr	0.48	0.49	0.45	0.45	0.44
Analyzed enzyme activities, U/kg					
Phytase	350	359	389	1,400	1,391
Xylanase	0	0	0	1,834	1,850

^*a*^PC, positive control diet; NC1, negative control diet 1; NC2, negative control diet 2; NC1 + E, negative control diet 1 plus multienzyme; and NC2 + E, negative control diet 2 plus multienzyme.

**Table 3.** Analyzed composition of phases 2–4 diets as-fed

	Diets^*a*^				
Item	PC	NC1	NC2	NC1 + E	NC2 + E
Phase 2					
Dry matter, %	86.4	90.8	87.9	87.8	88.1
Gross energy, kcal/kg	3,917	3,947	3,920	3,850	4,007
Crude protein, %	12.37	12.63	12.47	12.57	12.45
Neutral detergent fiber, %	11.13	13.02	13.29	13.23	14.75
Acid detergent fiber, %	4.57	6.12	5.67	4.96	6.32
Analyzed enzyme activities, U/kg					
Phytase	372	394	440	1,410	1,500
Xylanase	0	0	0	1,840	1,865
Phase 3					
Dry matter, %	88.5	88.4	88.3	88.7	88.2
Gross energy, kcal/kg	3,913	4,101	3,996	3,931	3,968
Crude protein, %	12.03	11.92	12.20	11.64	12.29
Neutral detergent fiber, %	12.76	16.23	15.97	13.71	15.91
Acid detergent fiber, %	5.53	5.64	7.31	5.64	5.78
Analyzed enzyme activities, U/kg					
Phytase	374	366	462	1,441	1,390
Xylanase	0	0	0	1,860	1,830
Phase 4					
Dry matter, %	88.5	88.4	88.3	88.7	88.2
Gross energy, kcal/kg	3,918	3,910	3,966	3,915	3,924
Crude protein, %	10.11	10.06	10.11	9.85	9.83
Neutral detergent fiber, %	12.27	14.94	16.04	16.44	17.79
Acid detergent fiber, %	4.62	6.45	7.7	6.51	8.22
Analyzed enzyme activities, U/kg					
Phytase	451	426	485	1,500	1,440
Xylanase	0	0	0	1,835	1,860

^*a*^PC, positive control diet; NC1, negative control diet 1; NC2, negative control diet 2; NC1 + E, negative control diet 1 plus multienzyme; and NC2 + E, negative control diet 2 plus multienzyme.

Data on effects of dietary treatment on growth performance, bone mineralization, AID of energy and nutrients, and ATTD of energy and nutrients are presented in Tables 4–7, respectively. Pigs fed PC diet had greater (*P* < 0.05) BW than those fed NC1 diet during Phases 1 and 2 of feeding, but not during Phases 3 and 4. The BW of pigs fed PC diet was greater (*P* < 0.05) than that of pigs fed NC2 diet during overall (34–120 kg BW) study period. Pigs fed PC diet had greater (*P* < 0.05) ADG than those fed NC1 diet during Phase 1, but not during Phases 2–4 of feeding. Also, the overall ADG of pigs fed PC diet did not differ from that of pigs fed NC1 diet. Pigs fed PC diet had greater (*P* < 0.05) ADG than those fed NC2 diet during the Phases 1, 2, and 4. Also, the overall ADG of pigs fed PC was greater (*P* < 0.05) than that of pigs fed NC2 diet. Pigs fed NC1 diet had greater (*P* < 0.05) ADFI than those fed PC diet during Phases 1 and 3 of feeding, but not during Phases 2 and 4; whereas pigs fed NC2 diet had greater (*P* < 0.05) ADFI than those fed PC diet during the Phase 3, but not during the Phases 1, 2, and 4. Pigs fed NC1 diet had lower (*P* < 0.05) G:F than those fed PC diet during the Phase 1 of feeding, but not during the Phases 2–4 and during the overall period. The G:F for NC2 diet was lower (*P* < 0.05) than that for the PC diet during the entire study period. The percent femur ash content for PC diet was greater (*P* < 0.05) than that for NC1 diet or NC2 diet. The femur breaking strength for PC diet was numerically, but not significantly greater than that for NC1 diet or NC2 diet. The PC, NC1, and NC2 diets did not differ in AID of GE, CP and indispensable AA. The ATTD of DM and GE for NC1 diet tended to be lower (*P* < 0.10) than that for the PC diet, whereas the ATTD of CP and P for NC1 diet was lower (*P* < 0.05) than that for PC diet. The ATTD of DM, GE, and P for the NC2 diet was lower (*P* < 0.05) than that for PC diet.

**Table 4.** Effect of dietary treatments on growth performance

	Diets^*a*^						*P-*value^*b*^			
Item	PC	NC1	NC2	NC1 + E	NC2 + E	SEM	Diet	NC	E	NC × E
BW, kg										
Day 0	34.61	34.60	34.60	34.67	34.54	-	-			
Phase 1	56.14^a^	53.83^b^	54.54^b^	56.03^a^	55.89^a^	0.472	0.004	0.341	<0.001	0.305
Phase 2	75.13^a^	72.77^b^	71.33^b^	74.81^a^	75.13^a^	0.670	0.001	0.754	<0.001	0.341
Phase 3	101.53^a^	99.68^ab^	98.12^b^	101.27^a^	100.41^ab^	1.000	0.092	0.264	0.021	0.883
Phase 4	124.45^a^	123.87^a^	118.90^b^	124.14^a^	123.26^a^	1.260	0.009	0.038	0.040	0.066
ADG, kg										
Phase 1	0.925^a^	0.821^b^	0.851^b^	0.912^a^	0.912^a^	0.021	0.004	0.301	<0.001	0.425
Phase 2	0.981^a^	0.936^ab^	0.884^b^	0.948^a^	0.980^a^	0.021	0.018	0.727	0.008	0.070
Phase 3	0.990	1.004	0.962	0.983	0.938	0.024	0.279	0.045	0.317	0.898
Phase 4	0.998^ab^	1.064^a^	0.902^c^	0.954^bc^	0.970^bc^	0.031	0.009	0.032	0.416	0.003
Overall	0.966^a^	0.955^a^	0.899^b^	0.955^a^	0.949^a^	0.013	0.008	0.022	0.164	0.046
ADFI, kg										
Phase 1	1.908^a^	2.312^b^	1.850^a^	1.896^a^	1.929^a^	0.038	<0.001	0.050	0.181	0.024
Phase 2	2.448	2.359	2.369	2.410	2.491	0.047	0.231	0.211	0.071	0.404
Phase 3	2.669^bc^	2.796^a^	2.819^ab^	2.647^bc^	2.621^c^	2.669	0.008	0.989	0.002	0.745
Phase 4	3.255^ab^	3.267^a^	3.076^ab^	3.022^b^	3.135^ab^	0.080	0.150	0.724	0.127	0.013
Overall	2.57^b^	2.70^a^	2.53^b^	2.48^b^	2.54^b^	0.04	0.003	0.353	0.096	0.045
G:F, kg/kg										
Phase 1	0.485^a^	0.367^c^	0.463^b^	0.480^ab^	0.474^ab^	0.007	<0.001	0.004	<0.001	0.002
Phase 2	0.401^a^	0.397^ab^	0.377^b^	0.394^ab^	0.394^ab^	0.007	0.099	0.151	0.237	0.321
Phase 3	0.372^a^	0.359^ab^	0.343^b^	0.373^a^	0.358^ab^	0.009	0.082	0.046	0.059	0.900
Phase 4	0.309^ab^	0.324^a^	0.295^b^	0.317^ab^	0.310^ab^	0.009	0.155	0.015	0.560	0.084
Overall	0.485^a^	0.367^c^	0.463^b^	0.480^ab^	0.474^ab^	0.007	<0.001	0.718	<0.001	0.130

^*a*^PC, positive control diet; NC1, negative control diet 1; NC2, negative control diet 2; NC1 + E, negative control diet 1 plus multienzyme; and NC2 + E, negative control diet 2 plus multienzyme.

^*b*^NC, main effects of negative control diet type; E, main effects of multienzyme; and NC × E, interaction between negative control diet type and multienzyme.

^abc^Within a row, means without a common superscript differ (*P* < 0.05).

**Table 5.** Effect of dietary treatment on bone mineralization

	Diets^*a*^						*P-*value^*b*^			
Item	PC	NC1	NC2	NC1 + E	NC2 + E	SEM	Diet	NC	E	NC × E
Femur ash, g	32.67^ab^	28.90^c^	29.78^bc^	35.97^a^	34.27^ab^	1.814	0.049	0.353	<0.001	0.118
Femur ash, %	55.32^a^	51.99^c^	52.75^bc^	54.33^ab^	55.15^a^	0.660	0.008	0.619	0.056	0.735
Femur breaking strength, N	2,513^bc^	2,191^c^	2,278^c^	2,950^a^	2,672^ab^	129.3	0.002	0.385	<0.001	0.177

^*a*^PC, positive control diet; NC1, negative control diet 1; NC2, negative control diet 2; NC1 + E, negative control diet 1 plus multienzyme; and NC2 + E, negative control diet 2 plus multienzyme.

^*b*^NC, main effects of negative control diet type; E, main effects of multienzyme; and NC × E, interaction between negative control diet type and multienzyme.

^abc^Within a row, means without a common superscript differ (*P* < 0.05).

**Table 6.** Effect of dietary treatment on AID of energy and nutrients

	Diets^*a*^						*P-*value^*b*^			
AID, %	PC	NC1	NC2	NC1 + E	NC2 + E	SEM	Diet	NC	E	NC × E
Dry matter	67.73^ab^	66.48^b^	66.21^b^	65.16^b^	71.73^a^	1.51	0.060	0.053	0.130	0.028
Gross energy	71.12^ab^	67.56^bc^	67.53^abc^	64.66^c^	73.43^a^	2.3	0.028	0.065	0.206	0.007
Crude protein	65.21^bc^	64.31^bc^	62.43^c^	69.53^ab^	75.14^a^	2.22	0.005	0.334	0.001	0.100
Indispensable AA										
Arg	80.01	81.14	81.51	81.47	84.60	1.47	0.264	0.105	0.306	0.299
His	74.47	77.25	76.69	75.63	79.34	1.74	0.319	0.351	0.818	0.208
Ile	71.98	73.76	73.72	74.15	77.59	2.23	0.472	0.433	0.403	0.424
Leu	75.39	75.88	75.50	76.90	78.99	1.87	0.622	0.594	0.307	0.482
Lys	77.28	78.36	77.99	79.47	82.50	2.08	0.404	0.627	0.188	0.462
Met	84.18^ab^	82.11^b^	82.01^b^	85.54^ab^	86.42^a^	1.26	0.048	0.478	0.006	0.454
Phe	73.63	74.69	74.30	75.71	78.81	2.01	0.378	0.333	0.252	0.272
Thr	65.37	67.04	66.40	67.24	73.81	2.97	0.232	0.129	0.333	0.120
Trp	76.53	77.06	76.68	77.11	80.25	2.45	0.770	0.627	0.457	0.484
Val	64.34	66.83	66.48	66.85	71.75	2.80	0.385	0.235	0.484	0.208
Dispensable AA										
Ala	67.05	68.86	67.49	68.98	72.71	2.42	0.482	0.596	0.329	0.290
Asp	70.83	72.54	71.41	72.27	75.19	2.51	0.764	0.885	0.448	0.498
Cys	64.17	63.10	64.24	63.50	69.93	3.16	0.519	0.363	0.327	0.483
Glu	80.07	80.87	81.87	82.11	85.21	1.71	0.277	0.527	0.166	0.543
Gly	36.06	47.25	54.26	54.35	58.48	6.58	0.102	0.422	0.366	0.779
Pro	76.35	72.49	76.06	77.10	79.57	2.22	0.321	0.143	0.173	0.299
Ser	69.08	70.77	69.48	70.74	74.98	2.54	0.461	0.500	0.370	0.250
Tyr	75.98	75.83	74.66	75.25	79.17	1.96	0.525	0.591	0.355	0.270

^*a*^PC, positive control diet; NC1, negative control diet 1; NC2, negative control diet 2; NC1 + E, negative control diet 1 plus multienzyme; and NC2 + E, negative control diet 2 plus multienzyme.

^*b*^NC, main effects of negative control diet type; E, main effects of multienzyme; and NC × E, interaction between negative control diet type and multienzyme.

^abc^Within a row, means without a common superscript differ (*P* < 0.05).

**Table 7.** Effect of dietary treatment on ATTD of energy and nutrients

	Diets^*a*^						*P-*value^*b*^			
ATTD, %	PC	NC1	NC2	NC1 + E	NC2 + E	SEM	Diet	NC	E	NC × E
Dry matter	75.62^ab^	73.79^b^	73.90^b^	75.76^ab^	76.43^a^	0.703	0.057	0.907	0.006	0.591
Gross energy	76.05^a^	74.32^ab^	73.65^b^	75.81^a^	75.67^a^	1.252	0.077	0.650	0.027	0.658
Crude protein	66.21^a^	62.70^b^	65.57^ab^	66.53^a^	67.13^a^	1.022	0.046	0.253	0.019	0.442
Neutral detergent fiber	69.91	71.57	74.61	70.47	75.52	1.074	0.002	<0.001	0.735	0.302
Acid detergent fiber	40.18^c^	43.18^bc^	48.90^b^	43.62^c^	53.91^a^	1.835	<0.001	<0.001	0.103	0.120
Ca	54.06^bc^	50.36^cd^	46.82^d^	55.20^b^	59.51^e^	1.381	<0.001	0.727	<0.001	0.010
P	38.19^b^	22.60^c^	27.51^c^	35.05^b^	48.56^b^	2.733	<0.001	0.003	<0.001	0.181

^*a*^PC, positive control diet; NC1, negative control diet 1; NC2, negative control diet 2; NC1 + E, negative control diet 1 plus multienzyme; and NC2 + E, negative control diet 2 plus multienzyme.

^*b*^NC, main effects of negative control diet type; E, main effects of multienzyme; and NC × E, interaction between negative control diet type and multienzyme.

^abc^Within a row, means without a common superscript differ (*P* < 0.05).

The NC diet type and multienzyme did not interact on BW during Phase 1, 2, and 3 of feeding. Multienzyme supplementation improved (*P* < 0.05) BW during phase 1–3 regardless of NC diet type. The NC diet type and multienzyme tended to interact (*P* = 0.066) on final BW (Phase 4) such that supplementation of NC1 diet with multienzyme did not affect the final BW, whereas supplementation of the NC2 diet increased (*P* < 0.05) the final BW of pigs. Multienzyme and NC diet type did not interact on ADG during Phase 1. However, multienzyme supplementation improved (*P* < 0.05) ADG during phase 1 regardless of NC diet type. The NC type and multienzyme tended to interact (*P* = 0.070) on ADG during Phase 2 such that supplementation of NC1 diet with multienzyme did not affect the ADG, whereas supplementation of the NC2 diet increased (*P* < 0.05) the ADG of pigs. The NC diet type and multienzyme interacted (*P* = 0.003) on ADG during Phase 4 such that supplementation of NC1 diet with multienzyme reduced (*P* < 0.05) the ADG, whereas supplementation of the NC2 diet tended to increase (*P* < 0.10) the ADG of pigs. The NC type and multienzyme interacted (*P* = 0.046) on the overall ADG such that supplementation of NC1 diet with multienzyme did not affect the ADG, whereas supplementation of the NC2 diet increased (*P* < 0.05) the ADG of pigs. The NC diet type and multienzyme interacted (*P* = 0.024) on the ADFI during the Phase 1 of feeding such that the ADFI for NC1 diet was reduced (*P* < 0.05), whereas that of the NC2 diet was unaffected by the multienzyme. Multienzyme supplementation reduced (*P* = 0.002) ADFI during Phase 3 regardless of NC diet type. The NC diet type and multienzyme interacted (*P* = 0.013) on ADFI during Phase 4 such that supplementation of NC1 diet reduced (*P* < 0.05) the ADFI, whereas the ADFI for NC2 diet was unaffected. The NC diet type and multienzyme interacted (*P* = 0.045) on the overall ADFI such that the ADFI for NC1 diet, but not of NC2 diet, was reduced (*P* < 0.05) by the supplementation. Multienzyme and NC diet type interacted (*P* = 0.002) on G:F during Phase 1 such that the supplementation increased (*P* < 0.05) G:F for NC1 diet and tended to increase (*P* < 0.10) G:F for NC2 diet. Also, multienzyme and NC diet type tended to interact (*P* = 0.084) on G:F during Phase 4 such that the multienzyme supplementation did not affect the G:F for NC1, but tended to increase (*P* < 0.10) G:F for NC2 diet. Multienzyme and NC diet type did not interact on overall G:F; however, the multienzyme increased (*P* < 0.001) overall G:F regardless of NC diet type. Multienzyme supplementation tended to increase (*P* = 0.056) percent femur ash content and increased (*P* < 0.001) bone breaking regardless of NC diet type. Multienzyme and NC diet type interacted (*P* = 0.007) on AID of GE such that multienzyme did not affect the AID of GE for NC1 diet and increased (*P* < 0.05) AID of GE for NC2 diet. Also, multienzyme and NC diet type tended to interact (*P* = 0.10) on AID of CP such that multienzyme supplementation tended to increase (*P* < 0.10) the AID of CP for NC1 diet, but increased (*P* < 0.05) AID of CP for NC2 diet. Multienzyme supplementation did not affect AID of all indispensable AA except of Met whose AID was increased (*P* = 0.006) by the supplementation regardless of the NC diet type. No interactions were detected between NC diet type and multienzyme on ATTD of DM, OM, GE, CP, NDF, ADF, and P. Multienzyme supplementation increased (*P* < 0.05) ATTD of DM, OM, GE, CP, Ca, and P.

## DISCUSSION

The ADG of pigs fed the NC1 diet was lower than that of pigs fed the PC diet during the first phase of feeding, implying that the reduction in NE, standardized ileal digestible AA, standardized total tract digestible P, and total Ca contents in PC diet by 74 kcal/kg, 3%, 43%, and 18%, respectively, was sufficient to reduce the growth performance of the pigs weighing between 34 and 55 kg. However, the ADG of pigs fed the NC1 diet did not differ from that of pigs fed the PC diet during the second, third and fourth phases of feeding, leading to lack of differences between NC1 and PC diets with regard to the overall ADG and final BW. Such results indicate that the reduction in NE, standardized ileal digestible AA, standardized total tract digestible P, and total Ca contents in PC diet by 74 kcal/kg, 3%, 43%, and 18%, respectively, during the phases of feeding 2, 3, and 4 was not sufficient to reduce the growth performance of the pigs, and could be attributed to an increase in digestive capacity of pigs with age. Similarly, Woyengo et al. (2008b) did not observe reduction in ADG of grow–finish pigs fed wheat-based diets due to reduction in DE value and available P contents in PC diet by 75 kcal/kg and 30.4%, respectively. Also, Li et al. (2012) did not observe reduction in ADG of pigs due to reduction in DE value by 50 kcal/kg. The ADG of pigs fed the NC2 diet was lower than that of pigs fed the PC diet during the first, second, and fourth phases of feeding. Also, the overall ADG and hence final BW of pigs fed the NC2 diet were lower than those of pigs fed the PC diet, implying that the reduction in NE, standardized ileal digestible AA, standardized total tract digestible P, and total Ca contents in PC diet by 124 kcal/kg, 5%, 43%, and 18%, respectively, was sufficient to reduce growth performance of the pigs. Emiola et al. (2009) observed reduction in ADG of growing pigs due to reduction in dietary DE by 124 kcal/kg. Jang et al. (2017) also served reduction in ADG of growing pigs due to reduction in dietary ME by 103 kcal/kg.

Supplementation of NC1 diet or NC2 diet with multienzyme increased ADG of pigs during the first phase (from 34 to 55 kg BW) of feeding, which was due to increased nutrient digestibility by the supplementation. This result is in accordance with several studies reporting an improvement of ADG in growing pigs fed diets supplemented with a multi-carbohydrase (Emiola et al., 2009; Kiarie et al., 2012; Ndou et al., 2015). Supplementation of NC2 diet with multienzyme increased ADG of pigs during the second phase of feeding (50–75 kg BW), and hence during the entire study period (34–125 kg BW), whereas it is not the case with NC1 diet. It should be noted that the NC2 diet was formulated to contain less NE and digestible AA than NC1 diet; and that the addition of multienzyme to NC2 diet resulted in an increase in AID of GE and CP, whereas the addition of multienzyme to NC1 diet only tended to increase in AID of CP. Also, the magnitude by multienzyme increased the ATTD of P for NC2 diet was greater than the magnitude by which it increased the ATTD of P for NC1 diet (21.1 vs. 12.5 percentage points). Additionally, overall ADFI for NC1 diet without multienzyme was greater than that for NC1 diet with multienzyme, whereas the overall ADFI for NC2 diet without multienzyme did not differ from that for NC1 diet with multienzyme. Thus, the greater effect of multienzyme on the NC2 diet than on NC1 diet with regard to ADG could be attributed to: 1) the fact that the NC1 diet was not so deficient in NE and digestible nutrient content as evidenced by the similarity between NC1 and PC diets in ADG of pigs, 2) greater increase in energy and nutrient digestibility for NC2 diet than for NC1 diet due to the supplemental multienzyme, and 3) greater ADFI and hence energy and nutrient intake for unsupplemented NC1 diet than for multienzyme supplemented NC1 diet. The results from the current study are in contrast to those from the study of Kiarie et al. (2012) who observed increased ADG of growing pigs from 22 to 55 kg BW, but not from 55 to 90 kg BW and hence for entire study period (22–90 kg BW) due to addition of a fiber-degrading enzyme product to corn–barley-based diet that had lower DE value than the recommended value by large magnitude (293 kcal/kg). Also, the results from the current study are in contrast to those from the study of Jang et al. (2017) who did not observe an increased ADG of growing pigs from 26 to 122 kg BW due to addition of fiber-degrading enzyme product to corn-based diet that had lower ME value than the recommended value by large magnitude (103 kcal/kg). However, the enzyme product used in the current study contained xylanase, β-glucanase, arabinofuranosidase, and phytase activities; whereas the enzyme product used in the studies of Kiarie et al, (2012) and Jang et al. (2017) contained only xylanase and β-glucanase activities, and xylanase activity, respectively. Thus, the differences between the current study and that of Kiarie et al. (2012) and Jang et al. (2017) with regard to the effects of supplemental multienzyme on growth performance of pigs could be attributed to differences in enzyme activities of the multienzyme products. As previously mentioned, arabinofuranosidase can de-branch arabinoxylans, leading to increased availability of xylan backbones for xylanase degradation. Also, phytase can hydrolyze phytic acid, leading to release of phytic acid-bound nutrients for digestion and absorption (Woyengo and Nyachoti, 2011). Multienzyme supplementation increased overall ADG and hence final BW for NC2 diet to that of the PC diet. Thus, the NE, standardized ileal digestible AA, total Ca, and standardized total tract digestible P contents in corn–wheat–wheat bran-based diets for pigs can be reduced by 124 kcal/kg, 5%, 18%, and 43%, respectively, without significant effects on growth performance of grow-finish pigs if the resulting low energy and nutrient diet is supplemented with the multienzyme that contain xylanase, β-glucanase, arabinofuranosidase, and phytase activities. Supplementation of the NC1 diet with multienzyme increased overall G:F, which was due to the reduction in ADFI by the supplementation. Supplementation of the NC2 diet with multienzyme increased overall G:F, which was due to the increase in ADG by the supplementation. Reason for the greater overall ADFI for NC1 diet without multienzyme than for NC1 diet with multienzyme is not clear.

The femur ash content and breaking strength for the PC diet significantly differ from those for NC1 diet or NC2 diet, which was partly due to the greater ATTD of P for the PC diet than for NC1 diet or NC2 diet. Supplementation of the NC1 diet or NC2 diet with multienzyme resulted in an increase in femur ash content and breaking strength, which could partly have been due to increase in P digestibility by the supplementation. Similarly, She et al. (2017) observed increased bone ash of weaned pigs due to dietary phytase supplementation. Woyengo et al. (2008a, 2010) also reported increased bone ash due to addition of phytase to diets of broilers. The femur ash content and breaking strength for the for multienzyme supplemented NC1 diet or multienzyme supplemented NC2 diet did not differ from those for the PC diet, implying the NE, standardized ileal digestible AA, Ca, and standardized total tract digestible P contents of the corn–wheat-based can be reduced by 124 kcal/kg, 5%, 18%, and 43%, respectively, without significant effect on bone mineralization if the resulting low energy and nutrient diet is supplemented with the enzyme product used in the current study.

The AID values of energy and nutrients were more variable than the ATTD values energy and nutrients, which could have due to the method (slaughter technique) of ileal digesta collection. Ileal digesta of pigs can be collected by slaughter technique or ileal cannulation technique (Nyachoti et al., 1997). The AID values obtained from ileal digesta that is collected by slaughter technique can variable due to diurnal variation in nutrient digestibility (Nyachoti et al., 1997). The ATTD of GE for the PC diet was greater than that for NC1 diet or NC2 diet, which was expected because the NC1 diet and NC2 diet were formulated to contain lower levels of NE than the PC diet. However, the AID of AA for the PC diet did not differ from that for NC1 diet or NC2 diet, and the reason for this similarity is not clear because the NC1 and NC2 diets were formulated to contain lower levels of digestible AA than the PC diet. It could have been due to the method that was used for ileal digesta collection. Multienzyme supplementation increased the AID of CP and methionine, and ATTD of GE and P for the NC1 and NC2 diets, which was due to hydrolysis of fiber by the NSPases and of phytate by phytase; as previously mentioned both NSPases and phytase were present in the multienzyme product used in the current study. In plant feedstuffs, phytate, and nutrients such as starch and AA are located within cells, whereas NSP are mainly found in cell walls (Woyengo and Nyachoti, 2011). Thus, NSPases can hydrolyze NSP in cell walls to increase the accessibility of phytase to phytate that is located within cells, thereby increasing the digestibility of phytate and availability of phytate-bound nutrients for digestion by gastric, pancreatic and small intestinal mucosa enzymes. Also, the hydrolysis NSP by NSPases can result in increased availability of the NSP-encapsulated nutrients for digestion by gastric, pancreatic, and small intestinal mucosa enzymes. Jang et al. (2017) also reported increased ATTD of DM and P due to supplementation of a combination of phytase and xylanase to corn-based diet for grow–finish pigs. Woyengo et al. (2010) reported increased digestibility of P and bone ash content of broilers due to addition of multienzyme that contained xylanase, β-glucanase, cellulose, and pectinase to phytase-supplemented diet. However, the magnitude of improvement in AID and ATTD of nutrients for NC2 diet was generally greater than that for NC1 diet. The NC2 diet was formulated to contain less NE and digestible AA than NC1 diet by partial replacement of corn and soybean meal in NC1 diet with wheat bran and soybean hulls. Wheat bran and soybean hulls are more fibrous than corn and soybean meal (NRC, 2012), implying that the former feedstuffs have greater content of substrate for the multienzyme than the latter. Thus, the greater effect of multienzyme on the NC2 diet than on NC1 diet with regard to AID and ATTD of nutrients could be attributed to the fact that the NC2 diet contained more enzyme substrate than NC1 diet. Zeng et al. (2018) similarly reported that supplementation of corn–wheat-based basal diet for growing pigs with enzyme product that contained galactanase, xylanase, mannanase, α-amylase, and cellulase activities did not improve nutrient digestibility when the basal diet did not contain wheat bran, but improved the AID of GE, NDF, and AA when the basal diet contained 20% wheat bran. Also, Zeng et al. (2018) observed that supplementation of corn–wheat-based basal diet for growing pigs with phytase improved AID of phytic acid regardless of whether or not the basal diet contained wheat bran, but the addition of an enzyme product that contained galactanase, xylanase, mannanase, α-amylase, and cellulose activities to phytase-supplemented diet improved AID of phytic acid only for wheat bran-containing basal diet.

The increased AID of CP observed in the current study due to multienzyme supplementation would indicate an increased digestibility of AA due to the supplementation. Nevertheless, methionine is the only AA whose AID was significantly improved by the multienzyme supplementation. In general, the increase in AA digestibility in response to enzyme supplementation is expected to be lower for highly digestible AA such as methionine than for less digestible AA like threonine (Cowieson, 2010). Thus, the results of the current study are contrary to the expectations. Nevertheless, a closer look at the change in AID of indispensable AA due to multienzyme supplementation reveal numerical increase in AID of most indispensable AA, and the magnitude of improvement of AID of some of these other indispensable AA is greater than for methionine. Among the indispensable AA, the SEM for AID of methionine was the lowest, implying that AID of the other indispensable AA was more variable than that of methionine. Thus, the statistically insignificant increase in AID of the other indispensable AA could have been due high variability in AID of these AA. However, it is not clear why the AID of AA other than methionine was more variable.

In conclusion, the overall ADG and ATTD of GE for the NC2 diet were lower than those for the PC diet, and multienzyme supplementation increased the overall ADG and ATTD of GE for the NC2 diet to those of the PC diet; the ATTD of P for multienzyme-supplemented NC2 diet was greater than that of PC diet. Thus, the NE and digestible AA and P can be lowered by ≤5% in multienzyme-supplemented diets without effects on growth performance pigs.
